# Supplementary material for: High-throughput Pore-C reveals the single-allele topology and cell type-specificity of 3D genome folding
Source: Nat Commun. 2023 Mar 6;14:1250. doi: 10.1038/s41467-023-36899-x (PMC9988853; doi:10.1038/s41467-023-36899-x)
Supplement: Supplementary file 1 — Supplementary Information [file 41467_2023_36899_MOESM1_ESM.pdf]

Supplementary Information

TABLE OF CONTENTS

Supplementary Figures

Supplementary Fig1. Solving nanopore clogging increases the output of multiway contact sequencing.

Supplementary Fig2. HiPore-C faithfully reproduces canonical 3D genome structures.

Supplementary Fig3. HiPore-C reveals interchromosomal chromatin interactions.

Supplementary Fig4. Multiway contacts spanning multiple compartments, TADs, and loops.

Supplementary Fig5. Comparison of HiPore-C adjacent (adj-) and nonadjacent (non-adj-) contacts.

Supplementary Fig6. Diversity and cell type-specificity of single-allele topology clusters underlying the formation of TADs.

Supplementary Fig7. HiPore-C reveals a cell type-specific enhancer hub at the β-globin locus.

Supplementary Fig8. HiPore-C reveals multi-promoter/enhancer interactions at the genome-wide scale.

Supplementary Fig9. HiPore-C reveals cell-type specific multiway promoter and enhancer interactions at the *HIST1* gene locus.

Supplementary Fig10. HiPore-C reveals cell-type specific multiway promoter and enhancer interactions in the MHC locus.

Supplementary Fig11. HiPore-C captures DNA methylation and chromatin topology simultaneously.

Supplementary Tables

Supplementary Table 1. The sequencing output of Pore-C. (related to Fig. 1a)

Supplementary Table 2. Cost of Hi-C, Pore-C and HiPore-C. (related to Fig.1 b and g)

Supplementary Table 3. Evaluation of optimized Pore-C library sequencing on an ONT MinION flow cell. (related to Supplementary Fig. 1b)

Supplementary Table 4. Statistics of sequencing output, alignment and pairwise contacts for each HiPore-C sequencing data. (related to Fig.1 and Supplementary Fig. 1)

Supplementary Table 5. Comparison between Pore-C and HiPore-C pipeline.

Supplementary Table 6. Distribution of tRNA genes with enriched interchromosomal interactions. (related to Supplementary Fig. 3d)

Supplementary Table 7. Statistics of CpG methylation in HiPore-C datasets.

Supplementary Data

Supplementary Data 1. Bin pairs signifcantly enriched interchromosomal interactions. (related to Fig. 3b)

Supplementary Data 2. The significant enrichment of interchromosomal interactions involving telomeres. (related to Fig.3c and Supplementary Fig. 3b)

Supplementary Data 3. The significant enrichment of interchromosomal interactions involving centromeres. (related to Fig.3e and Supplementary Fig. 3c)

Supplementary Data 4. Regional interchromosomal interaction enrichment and hub regions. (related to Supplementary Fig. 3f)

Supplementary Data 5. Multiway interaction frequency of gene promoters and enhancers in cell line GM12878. (related to Supplementary Fig. 8)

Supplementary Data 6. Multiway interaction frequency of gene promoters and enhancers in cell line K562. (related to Supplementary Fig. 8)

Supplementary Figures

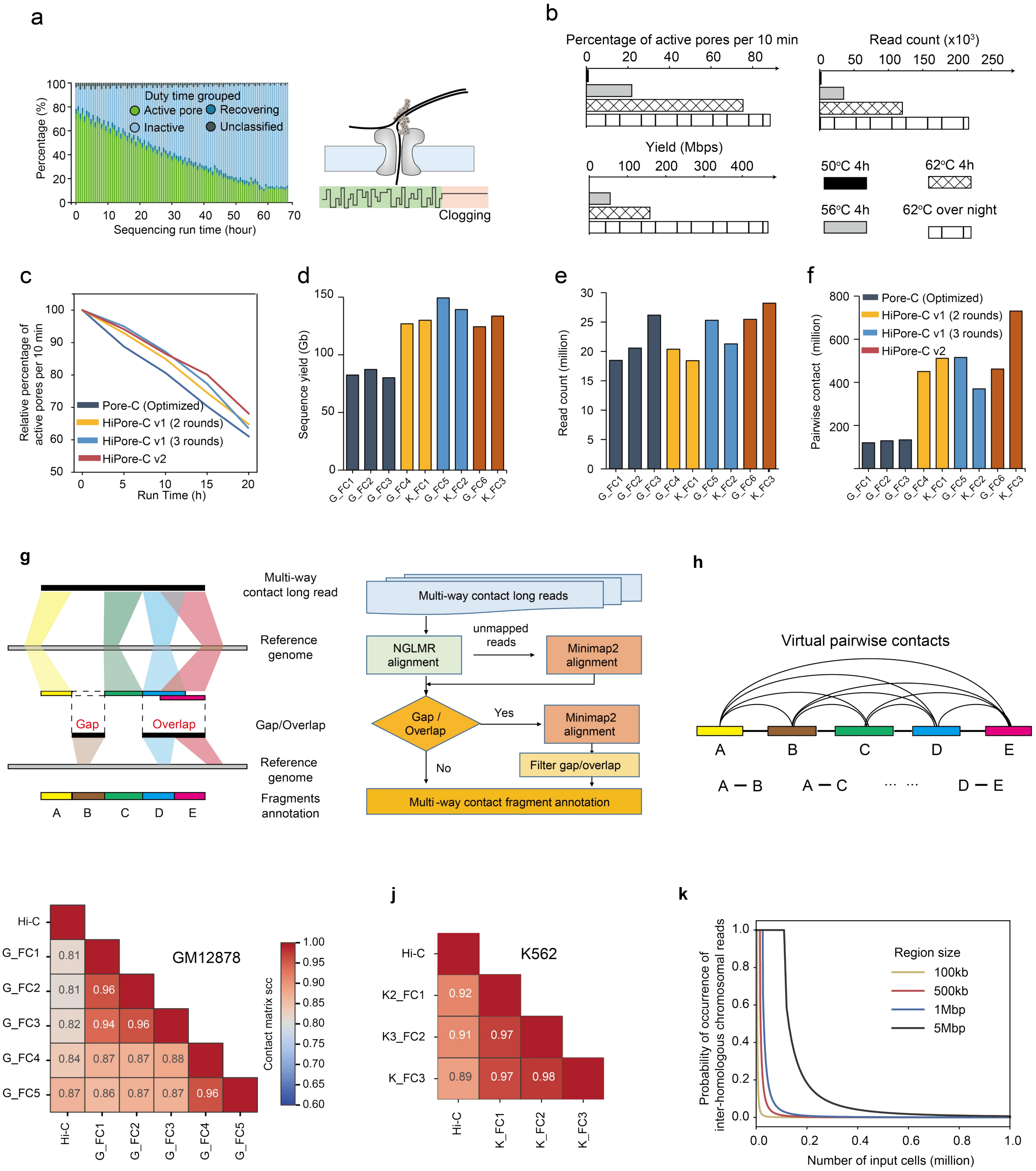

Supplementary Fig. 1 | Solving nanopore clogging increases the output of multiway contact sequencing.

a, Statistical report of the ONT nanopore channel states (active, inactive, recovering, and unclassified) during

Pore-C (optimized) DNA library sequencing. The diagram on the right shows the multiway contact DNA with peptide-DNA adducts clogging the flow cell channels during sequencing.

b, Evaluation of optimized Pore-C library sequencing on an ONT MinION flow cell. The percentage of active pores after 60 minutes sequencing, the number of generated reads, and total sequence yields were compared.

c-f, Comparison of the relative percentage of active pores per 10 minutes against sequencing run time, sequence yields, total reads per run, and virtual pairwise contact number for Pore-C (optimized, blue line), HiPore-C v1 (orange line for two rounds and cyan line for three rounds of reverse crosslinking and proteinase K digestion) and HiPore-C v2 (brown line).

g, Schematic workflow of MapPoreC.

h, Diagram showing the generation of virtual pairwise contacts from multiway contact reads. Reads with n ligated fragments contain C(n,2) (combination calculator) pairwise contacts. For example, reads with fragments A, B, C, D, and E, could generate ten pairwise contacts: A-B, A-C, A-D, A-E, B-C, B-D, B-E, C-D, C-E, and D-E.

i-j, Heatmap of Stratum-adjusted correlation coefficients between Hi-C datasets and replicates of HiPore-C on cell lines GM12878 (**i**) and K562(**j**). G_FC4-5 are HiPore-C replicates for GM12878, and K_FC1-3 are HiPore-C replicates for K562.

k, The occurrence probability of interhomologous chromosome reads. X-axis, input cell numbers in millions; y-axis, probability of observing at least two multi-way contact reads from homologous chromosome regions of a same cell. Yellow, red, blue, and black lines correspond to the region sizes of 100 kb, 500 kb, 1 Mb, and 5 Mb, respectively.

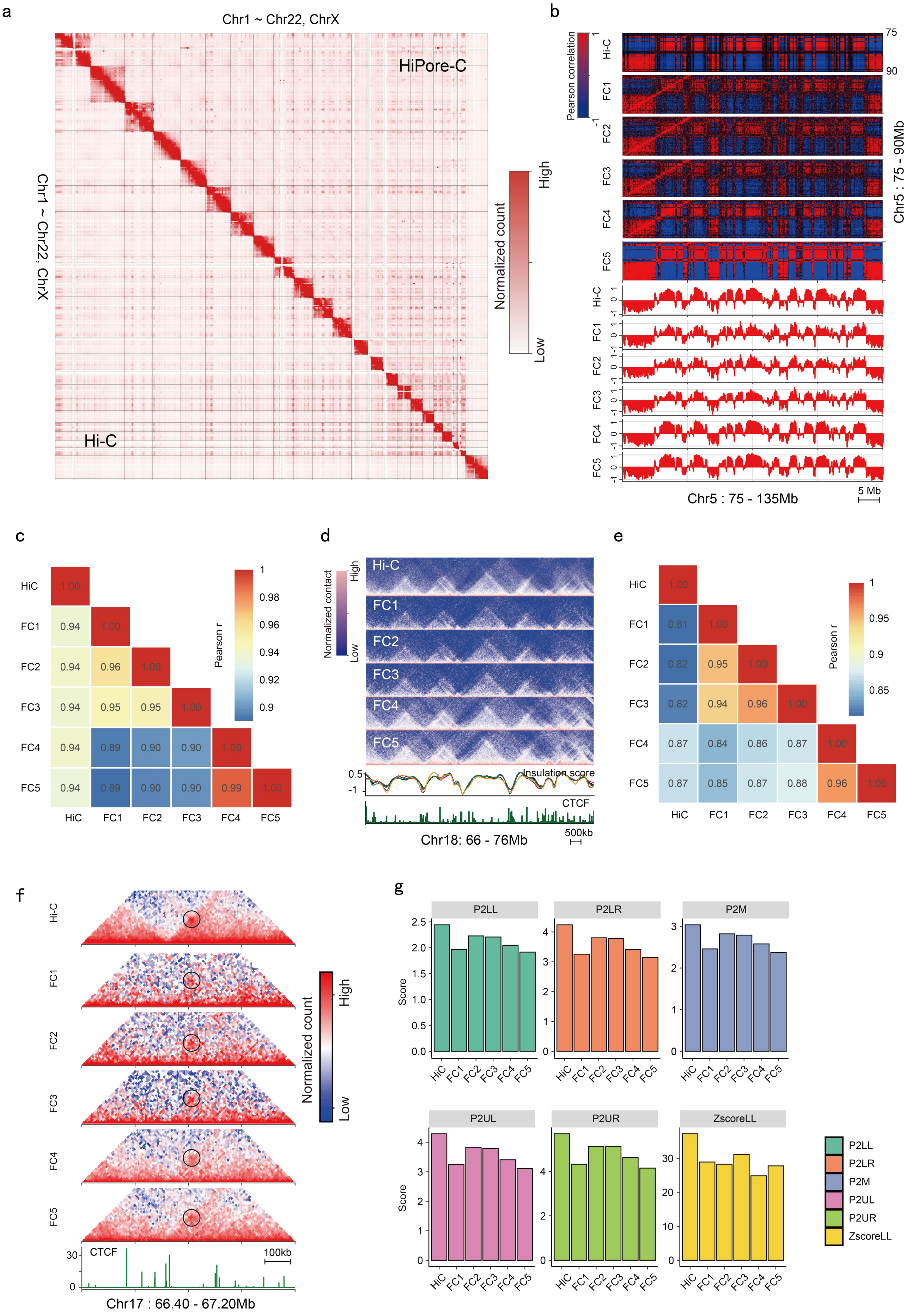

a
Chr1 ~ Chr22, ChrX
HiPore-C
Hi-C
Chr1 ~ Chr22, ChrX
Normalized count
High
Low
b
Pearson correlation
Hi-C
FC1
FC2
FC3
FC4
FC5
75
90
Chr5 : 75 - 90Mb
Chr5 : 75 - 135Mb
5 Mb
c
HiC
FC1
FC2
FC3
FC4
FC5
1.00
0.94
0.96
0.95
0.89
0.90
0.99
Pearson r
d
Normalized contact
High
Low
Hi-C
FC1
FC2
FC3
FC4
FC5
Insulation score
CTCF
Chr18: 66 - 76Mb
500kb
e
HiC
FC1
FC2
FC3
FC4
FC5
0.81
0.82
0.95
0.94
0.96
0.87
0.84
0.86
0.85
0.88
Pearson r
f
Hi-C
FC1
FC2
FC3
FC4
FC5
Normalized count
High
Low
CTCF
100kb
Chr17 : 66.40 - 67.20Mb
g
P2LL
P2LR
P2M
P2UL
P2UR
ZscoreLL
Score
HiC
FC1
FC2
FC3
FC4
FC5

Supplementary Fig. 2 | HiPore-C faithfully reproduces canonical 3D genome structures.

a, Comparison of global pairwise contact heatmaps between merged HiPore-C and Hi-C datasets (upper right, HiPore-C; bottom left, Hi-C).

b, An exemplary genomic region showing the reproducibility of compartments (100 kb resolution) with Hi-C as control. Eigenvector score tracks are shown below the correlation heatmaps.

c, Heatmap showing Pearson's correlation coefficients among eigenvector scores of HiPore-C replicates (FC1-5) and Hi-C data.

d, An exemplary genomic region showing the reproducibility of TADs (10 kb resolution) by HiPore-C with Hi-C data as control. The heatmap of normalized contacts are shown on top.

e, Histogram showing Pearson's correlation coefficients among insulation scores of HiPore-C replicates and Hi-C data.

f, Chromatin loops (10 kb resolution) revealed by HiPore-C and Hi-C contact heatmaps in an exemplary genomic region. The CTCF track is shown below the heatmaps.

g, HiPore-C-based APA results with Hi-C data as control. P2LL, the ratio of the central pixel to the mean of the pixels in the lower left corner; P2LR, the ratio of the central pixel to the mean of the pixels in the lower right corner; P2M, the ratio of the central pixel to the mean of the remaining pixels; P2UL, the ratio of the central pixel to the mean of the pixels in the upper left corner; ZscoreL, the Z score of the central pixel relative to all of the pixels in the lower left corner.

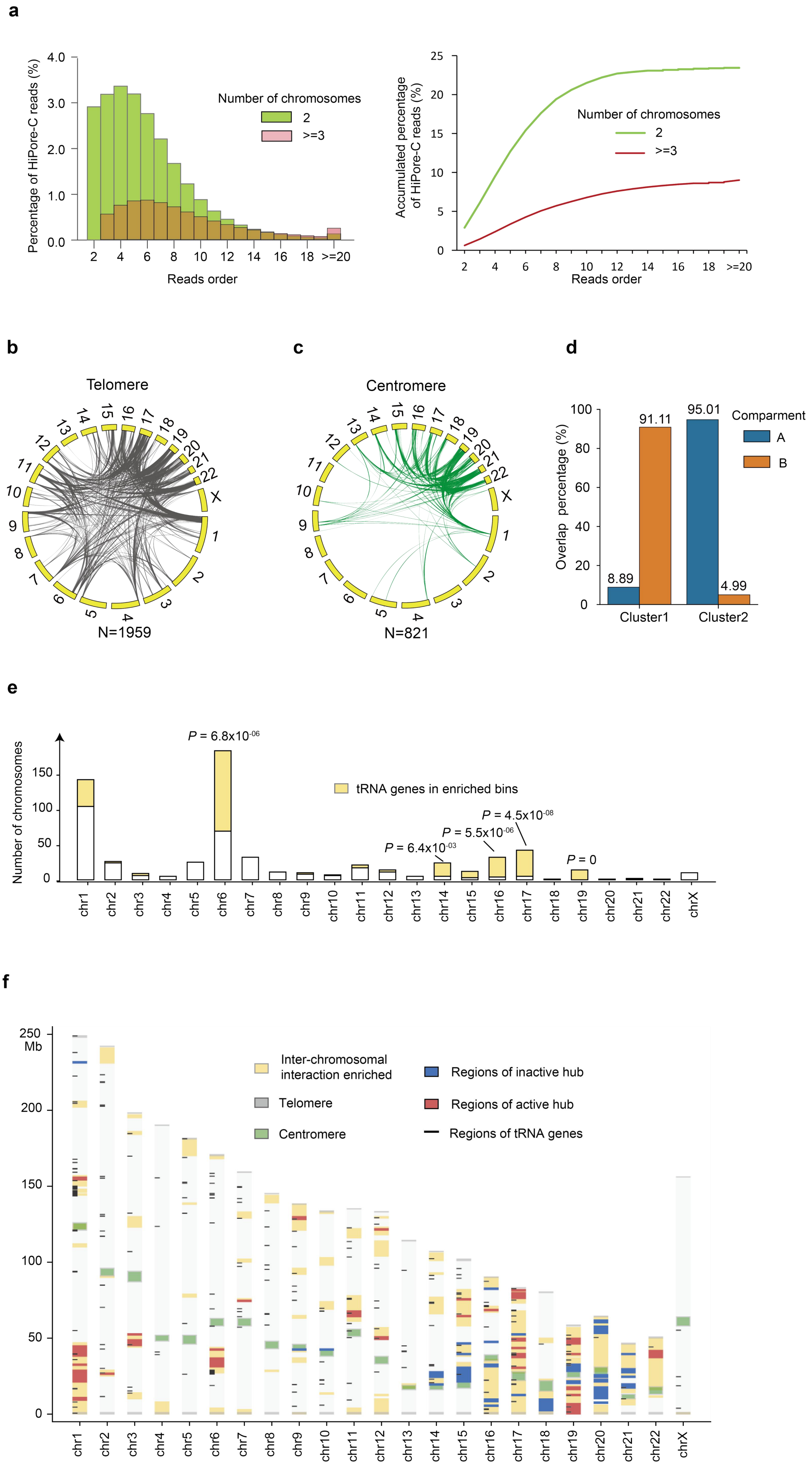

a
Percentage of HiPore-C reads (%)
Number of chromosomes
2
>=3
Reads order
Accumulated percentage of HiPore-C reads (%)
Number of chromosomes
2
>=3
Reads order
b
Telomere
N=1959
c
Centromere
N=821
d
Overlap percentage (%)
91.11
95.01
8.89
4.99
Comparment
A
B
Cluster1
Cluster2
e
Number of chromosomes
P = 6.8x10-06
tRNA genes in enriched bins
P = 4.5x10-08
P = 5.5x10-06
P = 6.4x10-03
P = 0
f
250 Mb
Inter-chromosomal interaction enriched
Telomere
Centromere
Regions of inactive hub
Regions of active hub
Regions of tRNA genes

Supplementary Fig. 3│HiPore-C reveals interchromosomal chromatin interactions.

a, Percentage (left) and accumulation ratio (right) of the multiway contact reads spanning two or more chromosomes. X-axis, the number of fragments in reads; y-axis, percentage or accumulated ratio of reads.

b and **c**, Circos diagrams of enriched interchromosomal contacts with one end in telomere or centromere regions.

d, The A/B compartment composition of the two cluster hubs.

e, Number of tRNA genes that are enriched in interchromosomal interactions. Yellow bars correspond to numbers of significantly enriched tRNA genes. Significance was calculated using a one-sided binomial test, and p-values were adjusted with Bonferroni's multiple corrections. Adjusted $P < 0.01$ was defined as significant. The number of tRNA genes, p-values and adjusted p-values of significant enriched tRNAs in different chromosomes were shown in Supplementary Table 6.

f, Diagram of regions significantly enriched in interchromosomal interactions. The colors gray, green, yellow, blue, red, and black indicate telomeres, centromeres, enriched genomic regions, regions in inactive hubs, regions in active hubs, and tRNA genes, respectively.

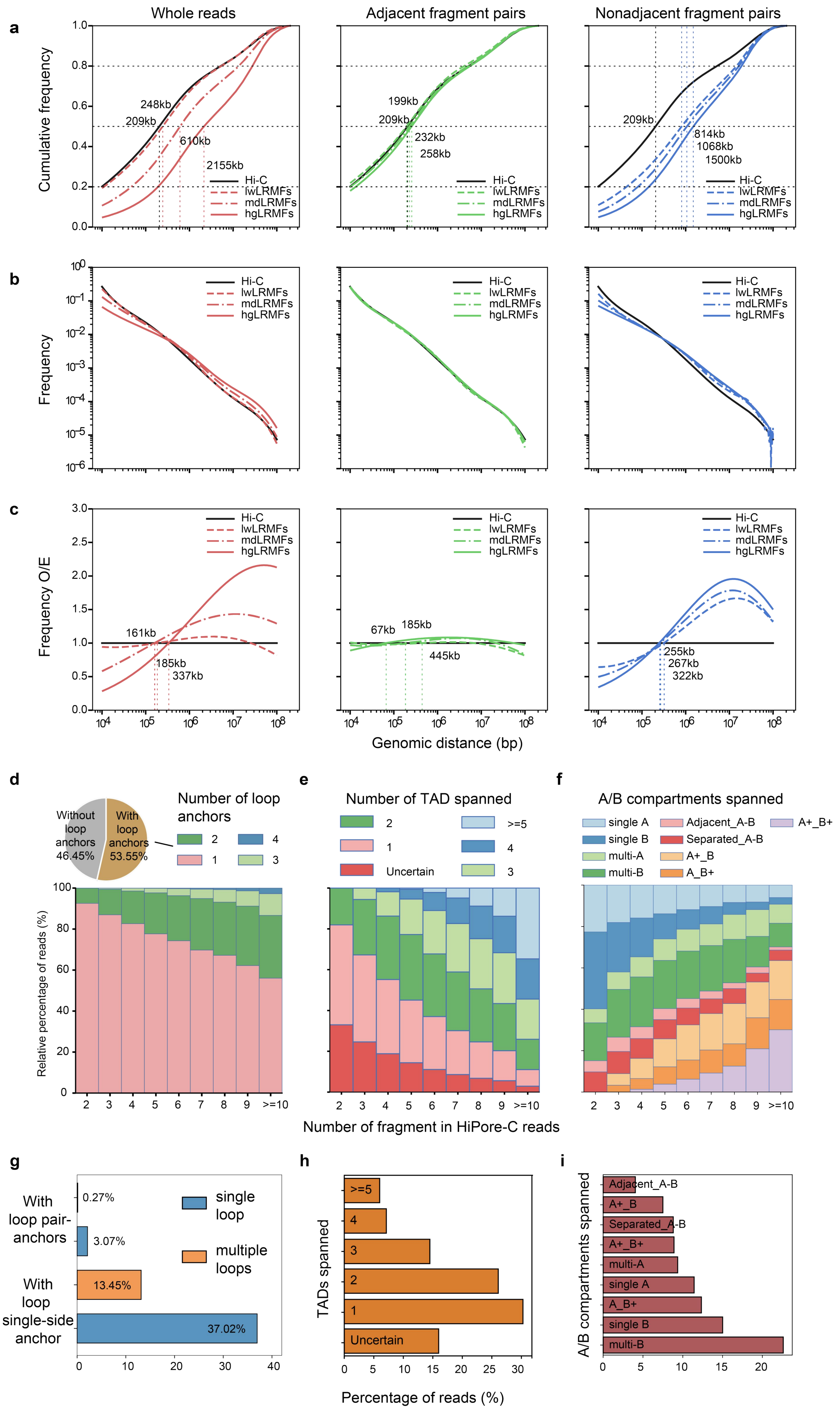

a
Whole reads
Adjacent fragment pairs
Nonadjacent fragment pairs
Cumulative frequency
248kb
209kb
610kb
2155kb
199kb
209kb
232kb
258kb
209kb
814kb
1068kb
1500kb
Hi-C
lwLRMFs
mdLRMFs
hgLRMFs
b
Frequency
c
Frequency O/E
161kb
185kb
337kb
67kb
185kb
445kb
255kb
267kb
322kb
Genomic distance (bp)
d
Without loop anchors 46.45%
With loop anchors 53.55%
Number of loop anchors
Relative percentage of reads (%)
e
Number of TAD spanned
Uncertain
f
A/B compartments spanned
single A
single B
multi-A
multi-B
Adjacent_A-B
Separated_A-B
A+_B
A_B+
A+_B+
Number of fragment in HiPore-C reads
g
With loop pair-anchors
With loop single-side anchor
0.27%
3.07%
13.45%
37.02%
single loop
multiple loops
h
TADs spanned
i
A/B compartments spanned
Percentage of reads (%)

Supplementary Fig. 4| Multiway contacts spanning multiple compartments, TADs, and loops.

a, Cumulative ratio of Hi-C and HiPore-C reads (left) and pairwise contacts between adjacent fragments (middle) and between nonadjacent fragments (right) in different groups of HiPore-C reads. HiPore-C reads are separated into three groups according to contained segment counts, which are lwLRMFs (2-3 fragments), mdLRMFs (4-9 fragments), and hgLRMFs (>=10 fragments).

b, Decaying curves of different types of paired fragments in different groups of HiPore-C reads.

c, Frequencies of different types of paired fragments in different groups of HiPore-C reads normalized against those of Hi-C data over continuous genomic distances.

d-f, Number of chromatin loop anchors (**d**), TADs (**e**), and compartments (**f**) spanned by multiway contact reads containing different numbers of fragments.

g-i, Percentage of multiway contact reads spanning different numbers of chromatin loop anchors (**g**), TADs (**h**), and compartments (**i**).

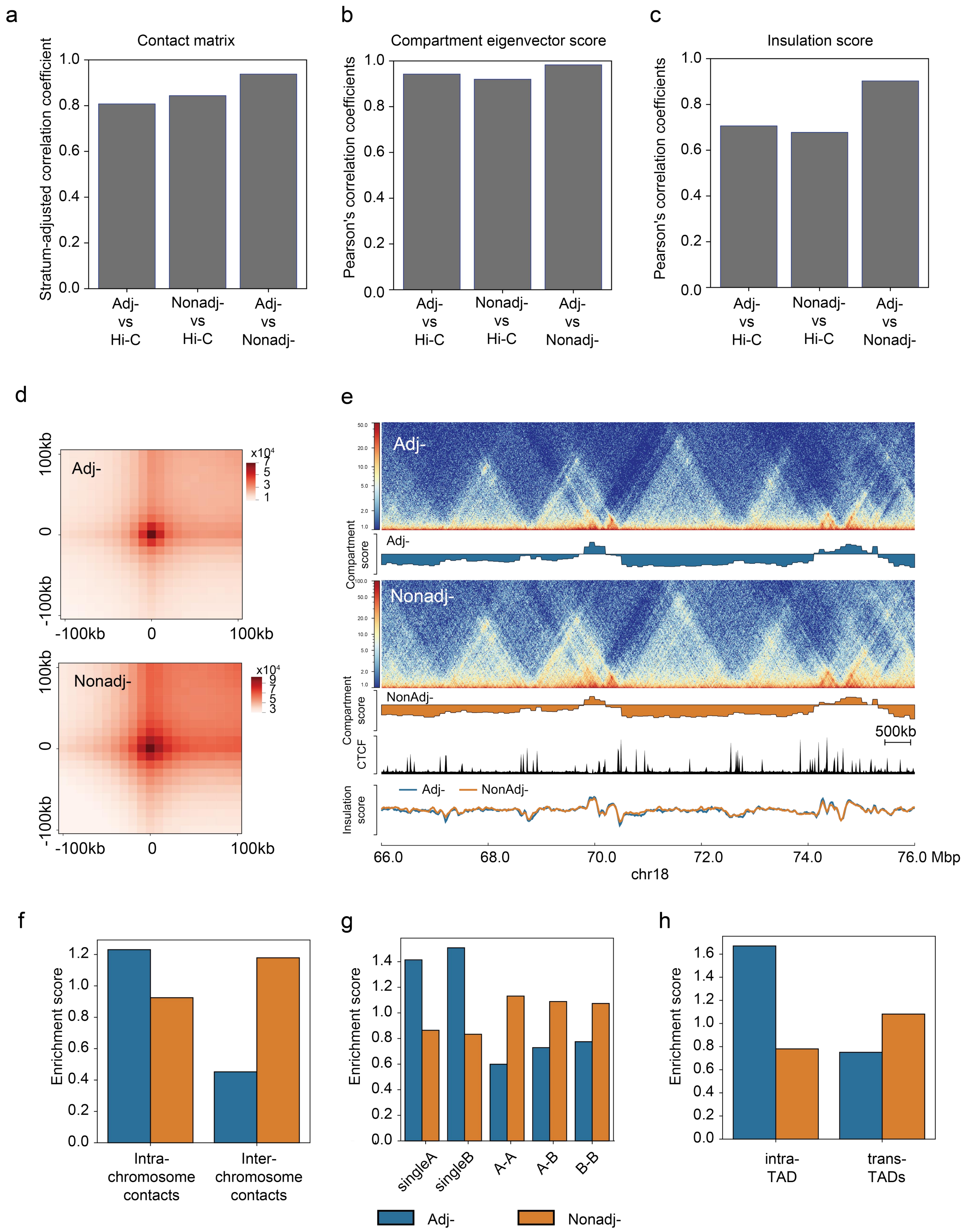

Supplementary Fig. 5|Comparison of HiPore-C adjacent (adj-) and nonadjacent (non-adj-) contacts.

a-c, The correlation coefficients of contact matrices (a), compartment eigenvector scores (b), and insulation scores (c) among HiPoreC adj-contacts, non-adj-contacts, and Hi-C contacts, respectively.

d, Comparison of the aggregated peaks between adj- and non-adj- contacts (10 kb resolution within 100 kb of loop anchors).

e, An exemplary region to show the similarity of TADs, compartments plotted using the datasets of adj-contacts and non-adj-contacts (10 kb resolution).

f-h, Enrichment analysis of intra- and inter-chromosome (f), compartment (g), and TAD (h) contacts in the datasets of adj- and non-adj-contacts. In panel g, A and B denote A and B compartments; singleA, singleB denote contacts that are present only inside a single A or a single B compartment; A-A, A-B, and B-B denote inter-compartment contacts.

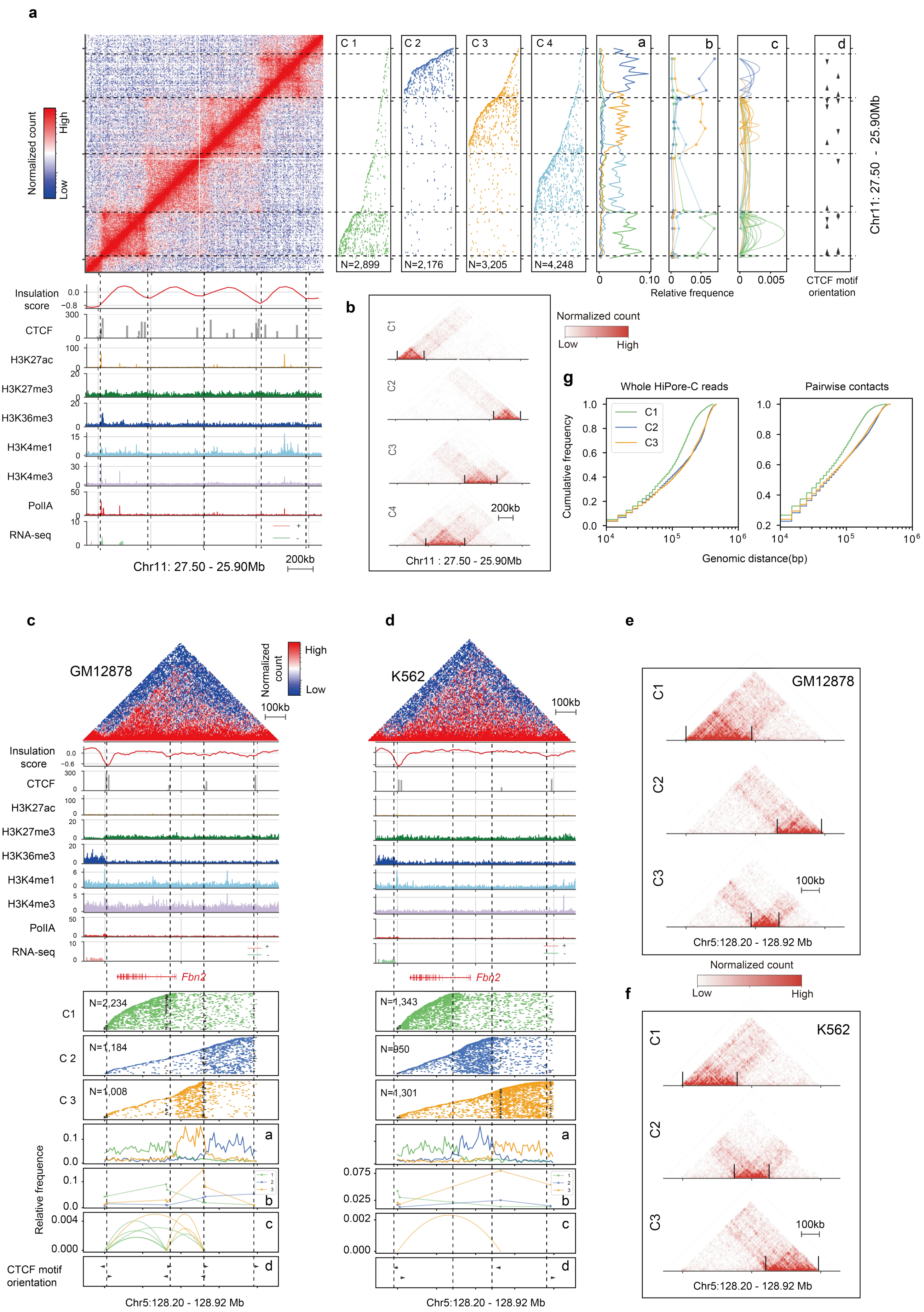

a
Normalized count
High
Low
C 1
C 2
C 3
C 4
N=2,899
N=2,176
N=3,205
N=4,248
0
0.10
0.05
0.005
Relative frequence
CTCF motif orientation
Chr11: 27.50 - 25.90Mb
Insulation score
CTCF
H3K27ac
H3K27me3
H3K36me3
H3K4me1
H3K4me3
PolIA
RNA-seq
200kb
b
Chr11 : 27.50 - 25.90Mb
g
Whole HiPore-C reads
Pairwise contacts
Cumulative frequency
Genomic distance(bp)
c
GM12878
d
K562
Fbn2
N=2,234
N=1,184
N=1,008
N=1,343
N=950
N=1,301
100kb
Chr5:128.20 - 128.92 Mb
e
f

Supplementary Fig. 6 | Diversity and cell type-specificity of single-allele topology clusters underlying the formation of TADs.

a, HiPore-C reads are clustered into four discrete genomic regions corresponding to four neighboring TADs in GM12878 cells. Five percent reads of each cluster were randomly selected and visualized in panels C1-C4.

b, Heatmaps generated from the four clusters of HiPore-C reads (**a**).

c and **d**, HiPore-C reads are clustered for the typical TAD containing the *fbn2* gene in GM12878 (**c**) and K562 (**d**) cell lines.

e and **f**, Heatmaps generated from the clusters of HiPore-C reads (**c** and **d**).

g, Cumulative frequency curves of the three clusters of HiPore-C reads (**c** and **d**) against genomic distance.

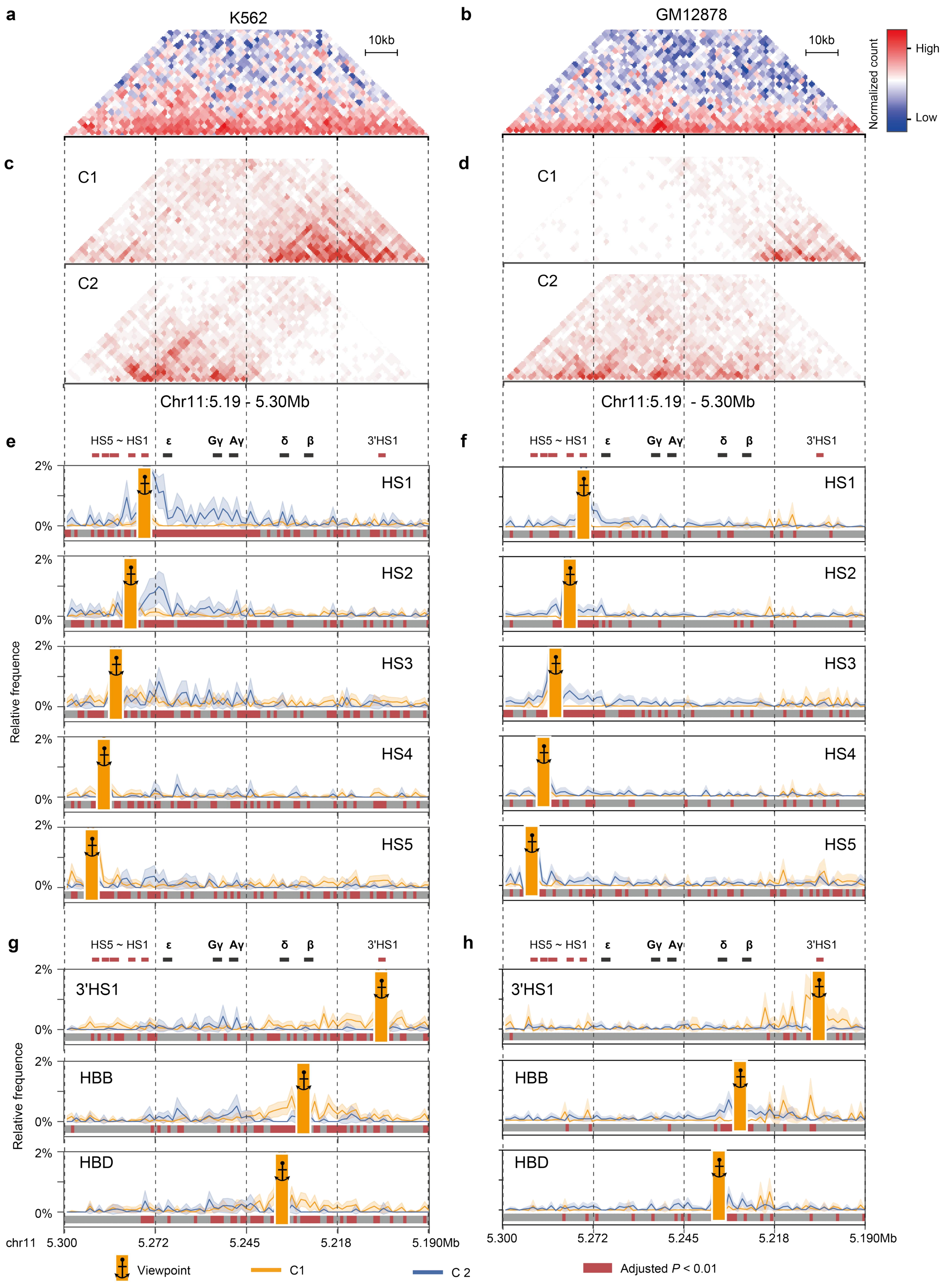

Supplementary Fig. 7 | HiPore-C reveals a cell type-specific enhancer hub at the β-globin locus.

a and **b**, Pairwise contact heatmaps of the human β-globin gene locus in K562 (**a**) and GM12878 (**b**) cell lines.

c and **d**, Heatmaps generated from clustered HiPore-C reads covering the human β-globin gene locus in K562 (**c**) and GM12878 (**d**) cell lines.

e and **f**, Multiway contacts anchored at different sites of the human β-globin gene locus (HS1, HS2, HS3, HS4, and HS5) in the two HiPore-C clusters in K562 (**e**) and GM12878 (**f**) cell lines. Viewpoints are shown as anchors. Reads from each cluster were randomly sampled 100 times to generate subsample sets. The relative appearance frequency of reads with viewpoints was calculated. Lines with shading represent the mean ± sd of the bin relative appearance frequency in the subsample sets. The statistical significance of the relative appearance frequency of bins was calculated by comparing the two clusters using a two-sided Welch's test with Bonferroni correction and is depicted with gray and dark red bars (gray, non-significant, adjusted $P >= 0.01$; red, significant, adjusted $P < 0.01$).

g and **h**, Multiway contacts anchored at different sites of the human β-globin gene locus (3'HS1 and β- and δ-globin genes) in the two HiPore-C clusters in K562 (**g**) and GM12878 (**h**) cell lines. The significance statistics and data presentation are same as in **e, f**.

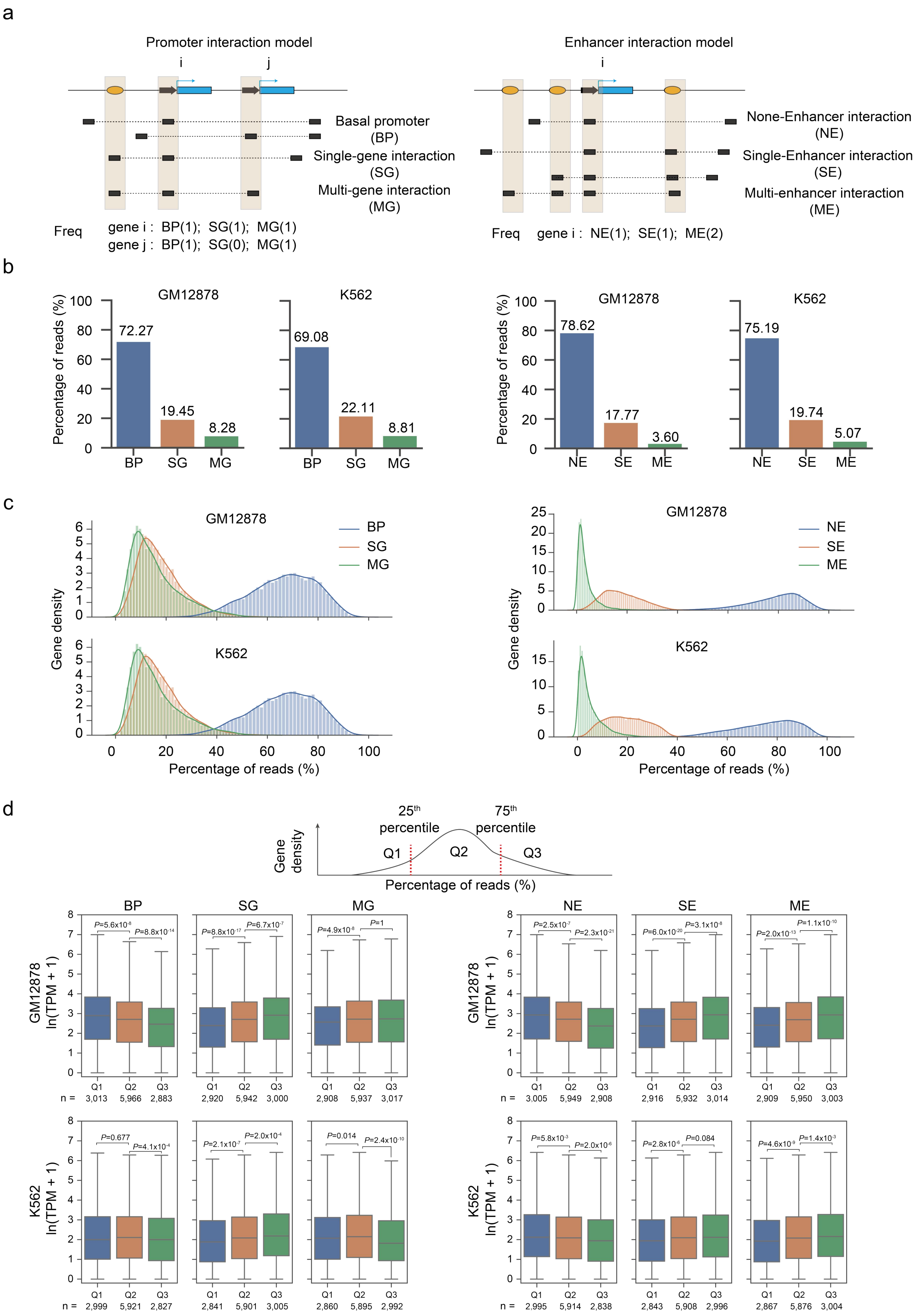
a
Promoter interaction model
i
j
Basal promoter
(BP)
Single-gene interaction
(SG)
Multi-gene interaction
(MG)
Freq
gene i : BP(1); SG(1); MG(1)
gene j : BP(1); SG(0); MG(1)
Enhancer interaction model
None-Enhancer interaction
(NE)
Single-Enhancer interaction
(SE)
Multi-enhancer interaction
(ME)
Freq gene i : NE(1); SE(1); ME(2)
b
GM12878
K562
Percentage of reads (%)
72.27
19.45
8.28
69.08
22.11
8.81
BP
SG
MG
78.62
17.77
3.60
75.19
19.74
5.07
NE
SE
ME
c
Gene density
Percentage of reads (%)
d
25th percentile
75th percentile
Q1
Q2
Q3
Gene density
Percentage of reads (%)
ln(TPM + 1)
P=5.6x10-8
P=8.8x10-14
n = 3,013 5,966 2,883
P=8.8x10-17
P=6.7x10-7
2,920 5,942 3,000
P=4.9x10-8
P=1
2,908 5,937 3,017
P=2.5x10-7
P=2.3x10-21
n = 3,005 5,949 2,908
P=6.0x10-20
P=3.1x10-8
2,916 5,932 3,014
P=2.0x10-13
P=1.1x10-10
2,909 5,950 3,003
P=0.677
P=4.1x10-4
n = 2,999 5,921 2,827
P=2.1x10-7
P=2.0x10-4
2,841 5,901 3,005
P=0.014
P=2.4x10-10
2,860 5,895 2,992
P=5.8x10-3
P=2.0x10-6
n = 2,995 5,914 2,838
P=2.8x10-6
P=0.084
2,843 5,908 2,996
P=4.6x10-5
P=1.4x10-3
2,867 5,876 3,004

Supplementary Fig. 8|HiPore-C reveals multi-promoter/enhancer interactions at the genome-wide scale.

a, Schematic showing interaction types in the promoter interaction model (left) and the enhancer interaction model (right). In promoters interaction model, there are Basal promoter (BP), Single-gene interaction (SG) and Multi-gene interaction (MG) reads. In enhancer interaction model, there are None-Enhancer interaction (NE) , Single-Enhancer interaction (SE) and Multi-Enhancer interaction (ME) reads. The interaction frequency and percentage of each genes were calculated according to gene promoter fragment.

b, Bar plots showing the proportions of HiPore-C reads classified into distinct interaction types in the promoter (left) and enhancer interaction models (right).

c, Histograms showing the gene density distribution across different type of promoter interaction (left) / enhancer interaction(right) frequencies. X-axis, the percentage of reads (%), represents the frequency of distinct interaction types of genes. Y-axis represents gene density.

d, Box plots showing the relationship between gene promoter (left) / enhancer (right) interaction frequencies and gene expression levels. Genes were divided into three groups showing low interaction frequency (Q1, 25th percentile), medium interaction frequency (Q2, 25th -75th percentile), and high interaction frequency (Q3, 75th percentile), respectively. The Kruskal-Wallis test, followed by Dunnet's test with Bonferroni correction was applied to calculate the statistical significance between each gene expression group. The center line, median; boxes, first and third quartiles; whiskers, 5th and 95th percentiles.

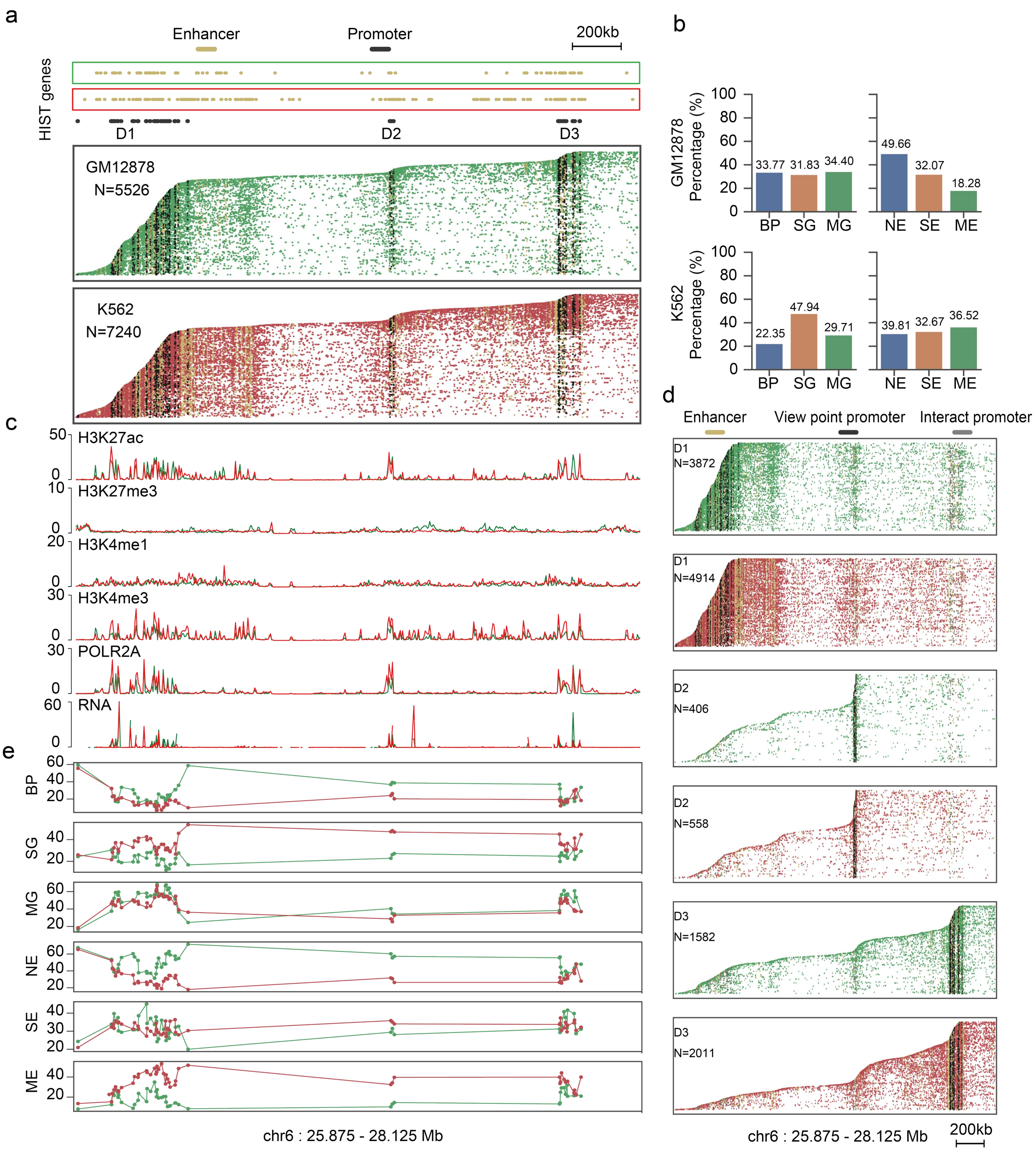

Supplementary Fig. 9|HiPore-C reveals cell-type specific multiway promoter and enhancer interactions at the *HIST1* gene locus.

a, HiPore-C reads covering the *HIST1* genes in GM12878 (green) and K562 (red) cell lines (2 kb resolution). We chose reads containing more than four fragments, with at least one fragment covering one *HIST1* gene promoter in the region of chr6:25.875-28.125 Mb to carry out the analysis. Gene promoters (black) and

enhancers (yellow) are shown in the top tracks. The promoters of 62 *HIST1* genes fall in 46 bins and are grouped in three clusters (D1, D2, D3) based on physical distances.

b, Bar plots showing the percentage of reads involved in distinct types of interaction in the promoter interaction model (left) and the enhancer interaction model (right).

c, Tracks showing the ChIP-seq (H3K27ac, H3K27me3, H3K4me1 and POLR2A) and RNA-seq signals in GM12878 (green) and K562 (red) cell lines.

d, HiPore-C reads anchored at different clusters (D1, D2, and D3) of *HIST1* genes showing multiway promoter/enhancer interactions within each cluster and across clusters.

e, Line plots comparing the interaction frequencies of *HIST1* genes in the GM12878 (green) and the K562 (red) cell lines.

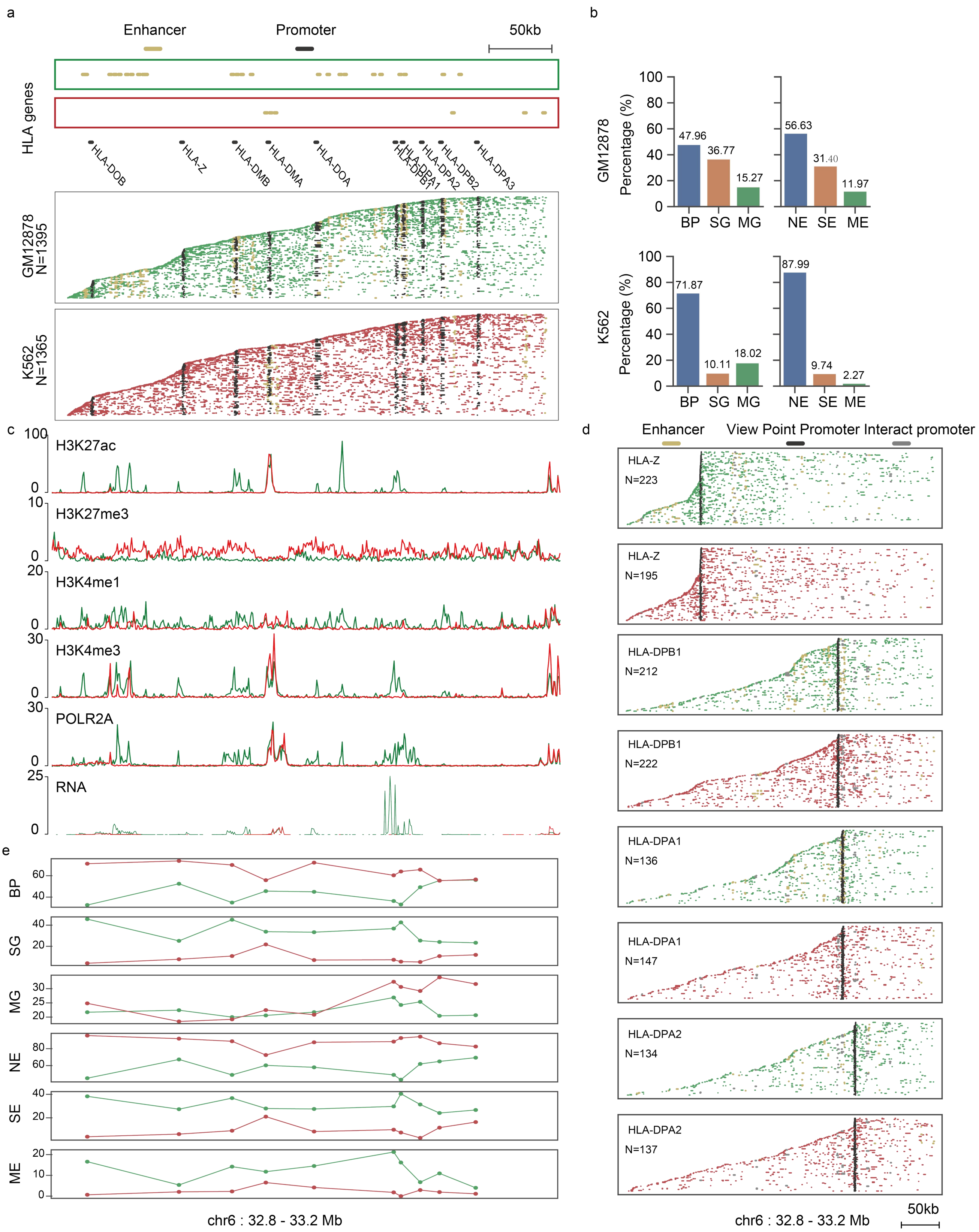

Supplementary Fig. 10|HiPore-C reveals cell-type specific multiway promoter and enhancer interactions in the MHC locus.

a, HiPore-C reads covering the MHC region in GM12878 (green and K562 (red) cell lines (2kb resolution). Only reads containing more than four fragments in the region of chr6:32.8-33.2 Mb and covering at least one *HLA* gene promoter fragment were used in this analysis. Gene promoters and enhancers are shown in the top tracks. *HLA* promoters were separated into ten bins.

b, Bar plots showing the percentage of reads involved in distinct types of interaction in the promoter interaction model (left) and the enhancer interaction model (right).

c, Tracks showing the ChIP-seq (H3K27ac, H3K27me3, H3K4me1 and POLR2A) and RNA-seq signals in GM12878 (green) and K562 (red) cell lines.

d, HiPore-C reads anchored at different *HLA* genes (*HLA-Z, HLA-DPB1, HLA-DPA1, HLA-DPA2*) showing multiway promoter/enhancer interactions across genes.

e, Line plots showing the different interaction frequencies of *HLA* genes in GM12878 (green) and K562 (red) cell lines.

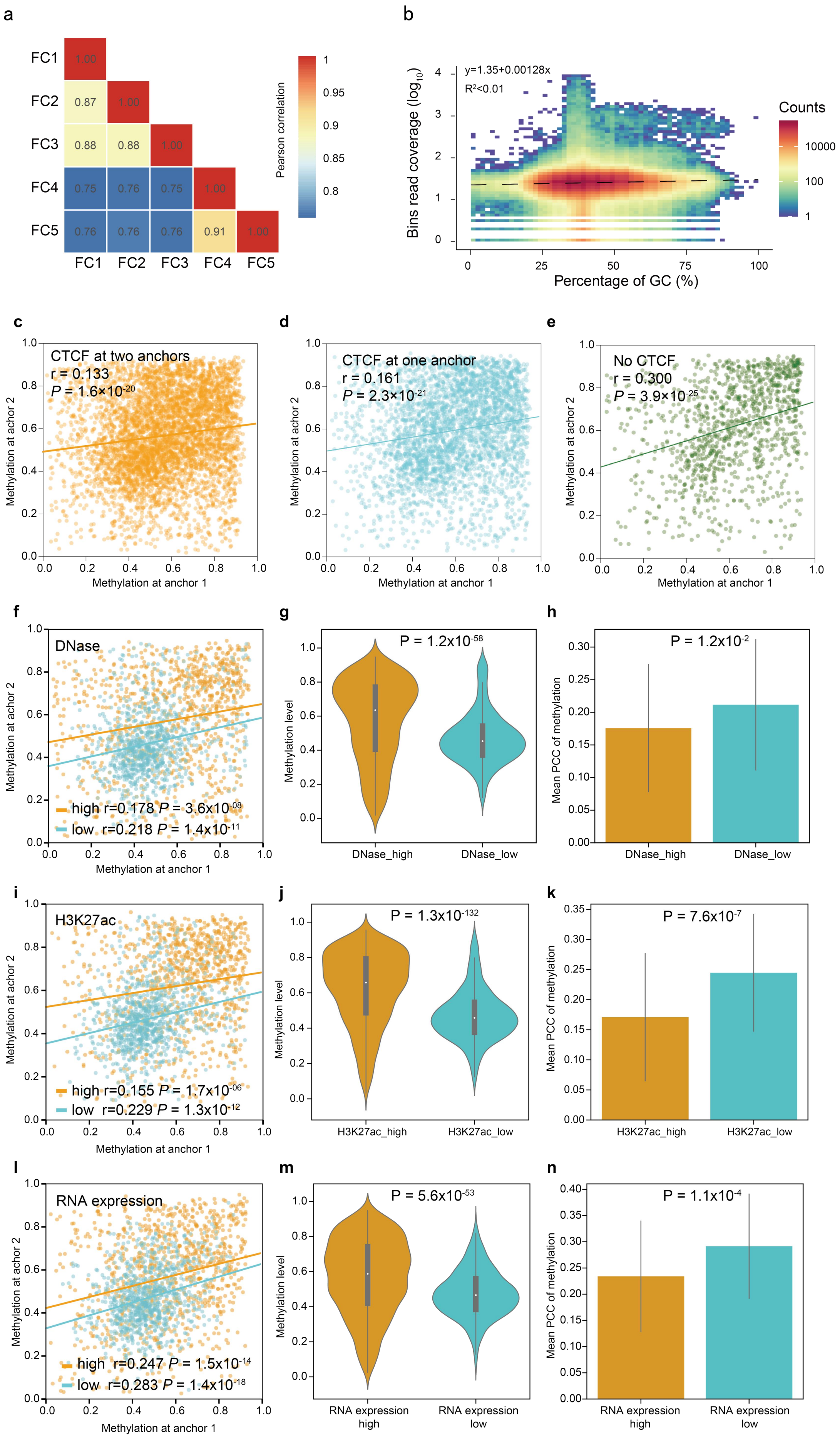
a
FC1
FC2
FC3
FC4
FC5
1.00
0.87
0.88
0.75
0.76
0.91
Pearson correlation
1
0.95
0.9
0.85
0.8
b
y=1.35+0.00128x
R²<0.01
Bins read coverage (log10)
Percentage of GC (%)
Counts
10000
100
1
c
CTCF at two anchors
r = 0.133
P = 1.6×10-20
Methylation at achor 2
Methylation at anchor 1
d
CTCF at one anchor
r = 0.161
P = 2.3×10-21
e
No CTCF
r = 0.300
P = 3.9×10-25
f
DNase
high r=0.178 P = 3.6x10-08
low r=0.218 P = 1.4x10-11
g
P = 1.2x10-58
Methylation level
DNase_high
DNase_low
h
P = 1.2x10-2
Mean PCC of methylation
i
H3K27ac
high r=0.155 P = 1.7x10-06
low r=0.229 P = 1.3x10-12
j
P = 1.3x10-132
H3K27ac_high
H3K27ac_low
k
P = 7.6x10-7
l
RNA expression
high r=0.247 P = 1.5x10-14
low r=0.283 P = 1.4x10-18
m
P = 5.6x10-53
RNA expression high
RNA expression low
n
P = 1.1x10-4

Supplementary Fig. 11│HiPore-C captures DNA methylation and chromatin topology simultaneously.

a, Histogram showing Pearson's correlation of CpG methylation with WGBS as a control.

b, Evaluation of HiPore-C coverage bias for sequences with different GC contents. The slope of the regression model represents the dependence degree of coverage on the GC content. The slopes of WGBS and WGS data obtained by ONT nanopore sequencing were reported to be -1.51 and -0.1[65], respectively.

c-e, Pair anchors' CpG methylation correlation at three loop types. **c**, Loops with CTCF at two anchors (n=4,858); **d**, Loops with CTCT at one anchor, (n=3,432); **e**, Loops with no CTCF (n=1,140).

f-h, Pearson's correlation coefficient(PCC) between paired anchors' CpG methylation and anchor methylation level distribution at chromatin loops with high- (n=943, labeled in orange) and low- density (n=937, labeled in cyan) of DNaseI-sensitive peaks. DNase-seq data was from Encode database. **f**, in dot plot of the pair anchors' methylation, linear regression trend line and Pearson's correlation coefficients are shown; **g,** in CpG methylation level distribution violin plot, the center dot, median; boxes, first and third quartiles; whiskers, 5th and 95th percentiles. **h**, Comparison of mean PCC between loops with high- and low- density.of DNAse peaks. Data are shown as the mean ± sd. Statistical significance was calculated by the two-sided unpaired Student's t-test both in **g** and **h** .

i-k, Analysis of CpG methylation in chromatin loops with high- (n=944, labeled in orange) and low- (n=934, labeled in cyan) density of H3K27ac peaks as in (**f-h**).

l-n, Analysis of CpG methylation in chromatin loops with high- (n=944, labeled in orange) and low- (n=931, labeled in cyan) signal level of RNA-seq as in (**f-h**).

Source	Sample	Enzyme	RunID	Platform	Read count (million)	Yield (Gb)	N50 read length(Kb)
Deshpande et.al. 2022	GM12878	DpnII	69689	PromethION	12.75	24.69	2.94
	HCC1954		e4b64		9.76	38.86	6.33
	HG002		9faf9		47.00	71.86	1.95
Data generated in this study	GM12878		G_7		15.29	65.98	5.35
	K562		K_4		15.49	78.96	6.54

Supplementary Table 1. The sequencing output of Pore-C.

Method	Sample ID	Platform	Cost($)	Sequence yield (Gb)	Generated reads(million)	Pairwise-contacts(million)	Costs per million pairwise contacts ($)
Pore-C (Deshpande et.al. 2022)	GM12878	Nanopore PromthION (1 flow cell)	1300	24.69	12.75	48.04	27.06
	HCC1954			38.86	9.76	160.38	8.11
	HG002			71.86	47.00	61.42	21.17
Pore-C (Control)	G_FC7			65.98	15.29	261.61	4.97
	K_FC4			78.96	15.49	436.82	2.98
Pore-C (Optimized)	G_FC1			82.45	18.48	119.95	10.84
	G_FC2			87.31	20.57	129.12	10.07
	G_FC3			80.11	26.18	133.51	9.74
HiPore-C v1 (2 rounds)	G_FC4			126.86	20.38	450.35	2.89
	K_FC1			129.95	18.43	511.87	2.54
HiPore-C v1 (3 rounds)	G_FC5			149.34	25.30	515.58	2.52
	K_FC2			139.25	21.29	369.95	3.51
HiPore-C v2	G_FC6			122.64	25.46	478.10	2.72
	K_FC3			133.60	28.21	731.05	1.78
Hi-C*	-	Illumina NovaSeq (1 lane)	4000	900	2700	1080	3.70

Supplementary Table 2. Cost of Hi-C, Pore-C and HiPore-C. The generation efficiency of pairwise-contact pairs from Hi-C reads was set to 40% based on previous experience.

Digestion temperature ℃	50	56	62	62
Reaction time	4h	4h	1h	Over Night
Number of active pores	9	511	410	511
Number of reads	9	29,055	120,125	215,182
Yield (Mbps)	0.0042	54.71	158.59	467.07
Pass reads (qual score >= 7)	0	28,831	111,668	205,588
Pass bases (Mbps)	0	54.48	148.64	448.52

Supplementary Table 3. Evaluation of optimized Pore-C library sequencing on an ONT MinION flow cell. Analysis result was from the first hour of MiniION flow cell sequencing.

Sample description	Protocol	**Pore-C**		**Pore-C (Optimized)**			**HiPore-C (2 times)**		**HiPore-C (3 times)**		**HiPore-C (Pronase)**	
	Sample ID	G_FC7	K_FC4	G_FC1	G_FC2	G_FC3	G_FC4	K_FC1	G_FC5	K_FC2	G_FC6	K_FC3
	Cell line	GM12878	K562	GM12878	GM12878	GM12878	GM12878	K562	GM12878	K562	GM12878	K562
**Sequence summary**	Reads generated (million)	15.29	15.49	18.48	20.57	26.18	20.38	18.43	25.30	21.29	25.46	28.21
	Sequence yield (Gb)	65.98	78.96	82.45	87.31	80.11	126.86	129.95	149.34	139.25	122.64	133.60
**Pass data**	Passed reads (million)	13.26	13.14	14.98	17.23	23.64	18.22	16.42	22.57	19.34	21.36	23.65
	Passed bases (Gb)	58.63	68.75	69.19	76.35	73.66	115.66	118.50	136.86	130.08	106.30	115.87
	Mean read length (Kb)	4.42	5.23	4.62	4.43	3.12	6.35	7.22	6.06	6.73	4.98	4.90
	N50 read length (Kb)	5.35	6.54	5.22	5.22	4.93	7.47	8.45	7.00	8.07	5.57	5.57
**Mapping and annotation**	Mapping reads (million)	12.55	12.46	14.97	17.22	23.61	18.16	15.29	22.50	19.31	21.64	23.65
	Mapping fragments (million)	68.99	88.00	57.73	63.78	70.07	118.29	92.44	141.77	110.93	133.68	168.46
	Mapping bases (Gb)	53.33	62.07	68.33	74.03	69.29	111.87	99.77	132.33	126.25	103.10	111.56
	Mean fragment number	5.50	7.06	3.86	3.70	2.97	6.51	6.05	6.30	5.74	6.18	7.12
	Median fragment number	4	6	3	3	2	6	6	5	5	6	7
	Mean fragment length(bp)	773.04	705.31	1183.74	1160.76	988.84	943.91	959.26	932.14	1130.39	764.52	655.33
	Median fragment length(bp)	547.00	511.00	834.00	820.00	699.00	653.00	662.00	649.00	765.00	771.00	459.00
**Fragments number distribution (million read count)**	1	1.15	0.73	2.03	2.83	7.89	1.31	0.88	1.41	1.62	1.24	1.65
	2	1.53	0.95	2.68	3.20	5.49	1.43	1.03	1.93	2.08	1.62	1.35
	3	1.83	1.28	2.85	3.20	3.52	1.73	1.32	2.35	2.43	2.11	1.63
	4	1.79	1.48	2.49	2.72	2.28	2.00	1.60	2.67	2.51	2.56	2.03
	5	1.50	1.44	1.86	2.01	1.48	2.09	1.75	2.72	2.31	2.74	2.37
	6	1.15	1.25	1.25	1.33	0.97	1.97	1.73	2.51	1.96	2.60	2.52
	7	0.86	1.03	0.77	0.82	0.64	1.70	1.57	2.12	1.57	2.24	2.43
	8	0.64	0.83	0.46	0.49	0.43	1.38	1.33	1.68	1.21	1.80	2.15
	9	0.48	0.66	0.26	0.28	0.29	1.07	1.09	1.28	0.91	1.37	1.79
	>=10	1.62	2.81	0.33	0.34	0.63	3.48	4.07	3.84	2.73	3.35	5.72
**Pair Contact Distribution**	Pairwise contacts count (million)	261.61	436.82	119.95	129.12	133.51	450.35	511.87	515.58	369.95	478.10	731.05
	Trans-contact (million)	170.28	276.16	23.92	26.71	28.19	138.25	140.76	161.88	88.65	181.29	316.94
	Cis-contact (million)	91.26	160.55	96.03	102.41	105.31	312.10	371.11	353.71	281.30	296.81	414.11

Supplementary Table 4. Statistics of sequencing output, alignment and pairwise contacts for each HiPore-C sequencing data.

Item	Pore-C pipeline (Deshpande et.al. 2022)	HiPore-C pipeline
Total reads (K)	100	
Total bases (Mbps)	461.32	
Mapping reads (K)	90.71	99.96
Mapping bases (Mbps)	443.13	461.25
Total Number of Mapping Fragments (K)	322.62	330.51
Total bases of Mapping Fragments (Mbps)	365.35	420.86
Mean Fragment length (bp)	1132.46	1273.37
Median Fragment length (bp)	953	966
Fragments per read	3.56	3.3
**Mapping gap and overlap**		
Gap ratio （gap bases / read_bases）	0.204	0.09
Overlap ratio (overlap bases / read_bases)	0.032	0.000163
Gap reads (gap percentage ≥0.1) (K)	61.3	27.97
Overlap reads (overlap ratio ≥0.1) (K)	10.64	0.002
**Mapping identity and integrity**		
Identity	0.777	0.816
Percentage of digestion match fragments (%)	63.69	83.36

Supplementary Table 5. Comparison between Pore-C and HiPore-C pipeline. After Guppy 4.5.3 fast mode base calling, 100,000 reads were randomly selected from the GM12878 datasets, and were aligned using Pore-C and HiPore-C pipeline respectively. The digestion match fragments defined as alignment fragments with both ends located within a distance less than 50bp to ends of the genome in-silicon restriction digested sites.

chrom	Number of tRNA genes	Interchromosome interaction enriched tRNA genes	p_value	adj_p_value
chr1	142	38	1.00E+00	1.00E+00
chr10	7	1	NA	NA
chr11	21	4	9.96E-01	1.00E+00
chr12	14	3	9.80E-01	1.00E+00
chr13	5	0	NA	NA
chr14	24	19	4.90E-04	6.37E-03
chr15	12	9	3.05E-02	3.97E-01
chr16	32	28	4.20E-07	5.46E-06
chr17	42	37	3.44E-09	4.48E-08
chr18	1	0		
chr19	14	14	0.00E+00	0.00E+00
chr2	26	2	1.00E+00	1.00E+00
chr20	1	1	NA	NA
chr21	2	1	NA	NA
chr22	1	1	NA	NA
chr3	9	3	NA	NA
chr4	5	0	NA	NA
chr5	25	0	1.00E+00	1.00E+00
chr6	183	114	5.20E-07	6.76E-06
chr7	32	0	1.00E+00	1.00E+00
chr8	11	0	1.00E+00	1.00E+00
chr9	10	2	NA	NA
chrX	10	0	NA	NA

S**upplementary Table 6. Distribution of tRNA genes with enriched interchromosomal interactions.** Significance was calculated using a one-sided binomial test, and p-values were adjusted with Bonferroni's multiple corrections.

Samples	Strand	Methylation CpGs	Unmethylation CpGs
G_FC1	+	165,798,021	117,346,424
	-	161,644,161	115,704,659
G_FC2	+	201,078,003	130,271,733
	-	196,485,463	128,749,919
G_FC3	+	176,758,744	122,248,676
	-	173,008,158	121,041,854
G_FC4	+	337,497,931	151,662,985
	-	330,741,578	150,066,761
G_FC5	+	395,431,637	183,084,012
	-	388,258,376	181,274,734

Supplementary Table 7. Statistics of CpG methylation in HiPore-C datasets.
