# Supplementary material for: High-throughput Pore-C reveals the single-allele topology and cell type-specificity of 3D genome folding
Source: Nat Commun. 2023 Mar 6;14:1250. doi: 10.1038/s41467-023-36899-x (PMC9988853; doi:10.1038/s41467-023-36899-x)
Supplement: Supplementary file 3 — Description of additional Supplementary File [file 41467_2023_36899_MOESM3_ESM.pdf]

## Descriptions of additional supplementary files

Supplementary Data 1. Bin pairs signifcantly enriched interchromosomal interactions.

Supplementary Data 2. The significant enrichment of interchromosomal interactions involving telomeres.

Supplementary Data 3. The significant enrichment of interchromosomal interactions involving centromeres.

Supplementary Data 4. Regional interchromosomal interaction enrichment and hub regions.

Supplementary Data 5. Multiway interaction frequency of gene promoters and enhancers in cell line GM12878.

Supplementary Data 6. Multiway interaction frequency of gene promoters and enhancers in cell line K562.
