# Supplementary material for: High-throughput Pore-C reveals the single-allele topology and cell type-specificity of 3D genome folding
Source: Nat Commun. 2023 Mar 6;14:1250. doi: 10.1038/s41467-023-36899-x (PMC9988853; doi:10.1038/s41467-023-36899-x)
Supplement: Supplementary file 10 — Reporting Summary [file 41467_2023_36899_MOESM10_ESM.pdf]

nature portfolio

Corresponding author(s): Chunhui Hou, Chuanle Xiao

Last updated by author(s): Jan 19, 2023

Reporting Summary

Nature Portfolio wishes to improve the reproducibility of the work that we publish. This form provides structure for consistency and transparency in reporting. For further information on Nature Portfolio policies, see our Editorial Policies and the Editorial Policy Checklist.

Statistics

For all statistical analyses, confirm that the following items are present in the figure legend, table legend, main text, or Methods section.

n/a	Confirmed	
☐	☒	The exact sample size (*n*) for each experimental group/condition, given as a discrete number and unit of measurement
☐	☒	A statement on whether measurements were taken from distinct samples or whether the same sample was measured repeatedly
☐	☒	The statistical test(s) used AND whether they are one- or two-sided<br>*Only common tests should be described solely by name; describe more complex techniques in the Methods section.*
☒	☐	A description of all covariates tested
☐	☒	A description of any assumptions or corrections, such as tests of normality and adjustment for multiple comparisons
☐	☒	A full description of the statistical parameters including central tendency (e.g. means) or other basic estimates (e.g. regression coefficient) AND variation (e.g. standard deviation) or associated estimates of uncertainty (e.g. confidence intervals)
☐	☒	For null hypothesis testing, the test statistic (e.g. *F*, *t*, *r*) with confidence intervals, effect sizes, degrees of freedom and *P* value noted<br>*Give P values as exact values whenever suitable.*
☒	☐	For Bayesian analysis, information on the choice of priors and Markov chain Monte Carlo settings
☐	☒	For hierarchical and complex designs, identification of the appropriate level for tests and full reporting of outcomes
☐	☒	Estimates of effect sizes (e.g. Cohen's *d*, Pearson's *r*), indicating how they were calculated

Our web collection on statistics for biologists contains articles on many of the points above.

Software and code

Policy information about availability of computer code

Data collection	No software was used for data collection.
Data analysis	This study utilized open software and packages, which are listed below: Python(3.7), R(3.3.1);Microsoft Excel 2016; Guppy (4.5.3); Megalodon (2.3.4); Nanoplot (1.30.1); ngmlr (0.2.7); minimap2 (2.17-r941); samtools (1.10); Pore-C-Snakemake pipeline (0.3.0); FIMO (5.3.3); cooltools (0.5); cooler (0.8.6); cocor (1.1-3, http://comparingcorrelations.org); HiCrep (1.2.0); Juicer(1.22.01); Juicebox(2.10.01); HiCExplorer (3.6); HiGlass(1.11.7) and FAN-C(0.9.23). And custom code and scripts generated within this project are available at https://github.com/zhengdafangyuan/HiPore-C.

For manuscripts utilizing custom algorithms or software that are central to the research but not yet described in published literature, software must be made available to editors and reviewers. We strongly encourage code deposition in a community repository (e.g. GitHub). See the Nature Portfolio guidelines for submitting code & software for further information.

Data

Policy information about availability of data

All manuscripts must include a data availability statement. This statement should provide the following information, where applicable:

- Accession codes, unique identifiers, or web links for publicly available datasets
- A description of any restrictions on data availability
- For clinical datasets or third party data, please ensure that the statement adheres to our policy

The HiPore-C data of GM12878 and K562 and processed files have been uploaded to GEO (https://www.ncbi.nlm.nih.gov/geo/) under series accession number (GSE202539). Processed files for the result analysis in this study are available from http://www.tgsbioinformatics.com/HiPore-C. Public Hi-C data listed in the manuscript are downloaded from 4DN Data Portal (https://data.4dnucleome.org). Public ChIP-seq, RNA-seq, DNAse-seq data listed in the manuscript are downloaded from ENCODE database (https://www.encodeproject.org).

Human research participants

Policy information about studies involving human research participants and Sex and Gender in Research.

Reporting on sex and gender	Sex and gender were not relevant to this study.
Population characteristics	Population chracteristics was not relevant to this study.
Recruitment	Recruitment was not relevant to this study.
Ethics oversight	Ethics oversight was not relevant to this study.

Note that full information on the approval of the study protocol must also be provided in the manuscript.

Field-specific reporting

Please select the one below that is the best fit for your research. If you are not sure, read the appropriate sections before making your selection.

☒ Life sciences ☐ Behavioural & social sciences ☐ Ecological, evolutionary & environmental sciences

For a reference copy of the document with all sections, see nature.com/documents/nr-reporting-summary-flat.pdf

Life sciences study design

All studies must disclose on these points even when the disclosure is negative.

Sample size	Pore-C experiments were performed on two cell lines, containing a total of five independent biological replicates. HiPore-C experiments were performed on two cell lines, each containing three independent biological replicates, to ensure the robustness of the results.
Data exclusions	No data were excluede.
Replication	Attempts at replication were successful. Each experiment conditions were performed on two cell lines, and these samples experiments were carried out with different preparations of cells, DNA and buffers. The results of data analysis showed good reproducibility.
Randomization	No randomization was performed. The sequencing experiments and analyses were carried on singleton cell lines.
Blinding	Blinding was not relevant to this study. The sequencing experiments and analyses were carried on singleton cell lines.

Reporting for specific materials, systems and methods

We require information from authors about some types of materials, experimental systems and methods used in many studies. Here, indicate whether each material, system or method listed is relevant to your study. If you are not sure if a list item applies to your research, read the appropriate section before selecting a response.

Materials & experimental systems

n/a	Involved in the study
☒	☐ Antibodies
☐	☒ Eukaryotic cell lines
☒	☐ Palaeontology and archaeology
☒	☐ Animals and other organisms
☒	☐ Clinical data
☒	☐ Dual use research of concern

Methods

n/a	Involved in the study
☒	☐ ChIP-seq
☒	☐ Flow cytometry
☒	☐ MRI-based neuroimaging

Eukaryotic cell lines

Policy information about cell lines and Sex and Gender in Research

Cell line source(s)	GM12878 was obtained from Coriell Institute and K562 cell line was purchased from ATCC (CCL-243).
Authentication	GM12878 was not authenticated but obtained directly from Coriell and used at low pass. K562 was not authenticated but obtained directly from ATCC and used at low pass.
Mycoplasma contamination	Not tested. The cell line was directly obtained from Coriell and ATCC, and used within 30 passages.
Commonly misidentified lines (See ICLAC register)	No commonly misidentified cell lines were used in this study.
